# The Tanzania Connect Project: a cluster-randomized trial of the child survival impact of adding paid community health workers to an existing facility-focused health system

**DOI:** 10.1186/1472-6963-13-S2-S6

**Published:** 2013-05-31

**Authors:** Kate Ramsey, Ahmed Hingora, Malick Kante, Elizabeth Jackson, Amon Exavery, Senga Pemba, Fatuma Manzi, Colin Baynes, Stephane Helleringer, James F Phillips

**Affiliations:** 1 Heilbrunn Department of Population and Family Health, Mailman School of Public Health, Columbia University, New York, USA; 2 Ifakara Health Institute, Dar es Salaam, Tanzania; 3 Tanzanian Training Center for International Health, Ifakara, Tanzania

## Abstract

**Background:**

Tanzania has been a pioneer in establishing community-level services, yet challenges remain in sustaining these systems and ensuring adequate human resource strategies. In particular, the added value of a cadre of professional community health workers is under debate. While Tanzania has the highest density of primary health care facilities in Africa, equitable access and quality of care remain a challenge. Utilization for many services proven to reduce child and maternal mortality is unacceptably low. Tanzanian policy initiatives have sought to address these problems by proposing expansion of community-based providers, but the Ministry of Health and Social Welfare (MoHSW ) lacks evidence that this merits national implementation. The Tanzania Connect Project is a randomized cluster trial located in three rural districts with a population of roughly 360,000 ( Kilombero, Rufiji, and Ulanga).

**Description of intervention:**

Connect aims to test whether introducing a community health worker into a general program of health systems strengthening and referral improvement will reduce child mortality, improve access to services, expand utilization, and alter reproductive, maternal, newborn and child health seeking behavior; thereby accelerating progress towards Millennium Development Goals 4 and 5. Connect has introduced a new cadre — Community Health Agents (CHA) — who were recruited from and work in their communities. To support the CHA, Connect developed supervisory systems, launched information and monitoring operations, and implemented logistics support for integration with existing district and village operations. In addition, Connect’s district-wide emergency referral strengthening intervention includes clinical and operational improvements.

**Evaluation design:**

Designed as a community-based cluster-randomized trial, CHA were randomly assigned to 50 of the 101 villages within the Health and Demographic Surveillance System (HDSS) in the three study districts. To garner detailed information on household characteristics, behaviors, and service exposure, a random sub-sample survey of 3,300 women of reproductive age will be conducted at the baseline and endline. The referral system intervention will use baseline, midline, and endline facility-based data to assess systemic changes. Implementation and impact research of Connect will assess whether and how the presence of the CHA at village level provides added life-saving value to the health system.

**Discussion:**

Global commitment to launching community-based primary health care has accelerated in recent years, with much of the implementation focused on Africa. Despite extensive investment, no program has been guided by a truly experimental study. Connect will not only address Tanzania’s need for policy and operational research, it will bridge a critical international knowledge gap concerning the added value of salaried professional community health workers in the context of a high density of fixed facilities.

Trial registration: ISRCTN96819844

## Background

**Figure 1:**
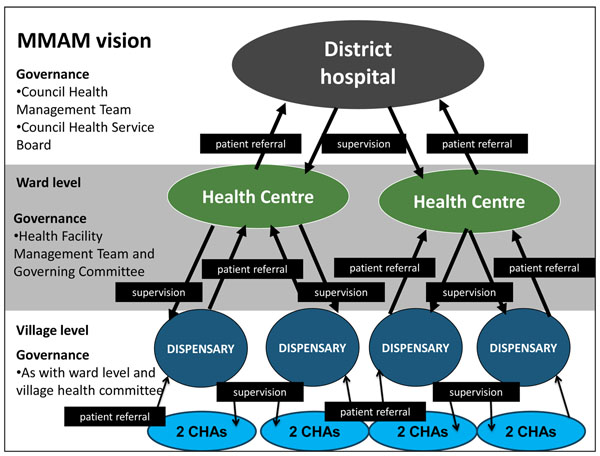
**District health system in Tanzania based on MMAM vision.** The figure shows the levels of care and interactions at the district health system level as envisioned in the MMAM policy in addition to corresponding governance structures at each level. The Connect project focuses on the CHA at the community level as well as linking the various levels.

Tanzania pioneered many aspects of primary health care that were later endorsed at the 1978 conference on International Primary Health Care in Almaty (formerly Alma-Alta), Kazakhstan, through the Alma Ata Declaration. Plans in the early 1970s envisaged the rapid development of rural health centers and dispensaries providing primary health care services as well as launching of a Village Health Worker (VHW) Program [1]. VHWs were intended to extend services to villages, particularly due to the recognition that establishing dispensaries in every village was not immediately feasible [2]. While groundbreaking, these programs encountered challenges in ensuring uniform implementation nationwide and with retention and effectiveness related to provision of systemic support and adequate remuneration [3]. Revitalized in the 1980s with support from child-survival programs, early problems were partly addressed, but the programs were limited by their project-based nature, lack of formalized support systems, and reliance on local government mechanisms for remuneration. By the 1990s and early 2000s, despite the admirable continuing dedication of numerous VHW, many were working as assistants to facility-based providers and were largely maintained through periodic training by vertical health programs; the primary health care component of their work was largely unsupported [4,5]. Due to the prolonged investment in rural health infrastructure, Tanzania now maintains one of the highest densities of health facilities amongst African countries. Health service organization follows the standard pyramid of dispensaries, health centers, and hospitals (Figure 1).

Connect focuses on the community level as well as linking the various levels through referral and feedback.

Household distance to the nearest health center is, on average, 10 kilometres (5 kilometres for dispensaries), but quality of care is often poor, with human resources operating at 32% of the required skilled workforce, insufficient medical equipment, and chronic shortages of medicines and supplies [6]. This is also undermined by weak referral systems that do not adequately identify and respond to acute conditions [7]. Consequently, availability, access, and utilization of essential maternal newborn child health (MNCH) services remain variable across the country.

While Tanzania has made substantial progress in the reduction of child mortality and is likely on track to reach MDG 4 [8], inequities remain with under-5 mortality rates ranging from 84 per 1,000 live births for the wealthiest quintile to 103 per 1,000 for the lowest quintile [8]. In addition, limited progress has been observed in reducing neonatal and maternal mortality, with neonatal mortality now comprising 30% of child mortality [9]. Care seeking for child illness was 57% for acute respiratory infection symptoms or fever and 47% for diarrhea, with declines in appropriate treatment of under-5 malaria and diarrhea in the last five years. Of children with fever symptoms who received anti-malarials within 24 hours, the rates varied from almost 50% for the wealthiest quintile to as low as 36.5% for the lowest quintile. Use of modern contraception (27%) has shown only modest gains in the last five years, with rates of approximately 35% among urban populations as compared to 25.5% among rural. For antenatal care, coverage is near universal at 98% for at least one antenatal care visit but drops significantly to 43% for four visits or more. Women also report late for the first visit, at a median of 5.4 months gestation. Only about half of Tanzanian women delivered in health facilities in 2010 [10]. Postnatal care remains low with 35% of women having received a postnatal check-up within six weeks after delivery.

Research has identified interventions that can reduce maternal, newborn, and child mortality as components of integrated primary care [11-16]. Growing evidence shows that many of these interventions can be effectively delivered by community health workers or “lay health workers” at the community level [17,18]. The Navrongo experiment in Northern Ghana provides an example of the mortality reductions that can be achieved in a relatively short time with the right package of interventions provided at community level [19,20]. Effectiveness of community health workers in increasing preventive behaviors to improve newborn survival has been demonstrated in Asia [21]. The ability of community health workers to correctly diagnose and treat conditions such as malaria, acute respiratory infections, and diarrhea using integrated management of childhood illness (IMCI) has been established in a number of settings in Africa and Asia [22-24]. Household visits by community health workers, including pregnancy monitoring and counseling, have also been shown to increase utilization of antenatal care, skilled delivery care, and immunization services [17,25]. Additionally, it is expected that community health workers will have greater cultural competence than facility-based health worker cadres [7,24].

Although the evidence-base is expanding for establishing the plausibility of community health worker impact, direct trial of the added value of paid community health workers remains the subject of discussion and debate. While there is widespread investment in community-based primary health care, all impact studies have been plausibility trials rather than randomized trials [5,23,26]. Moreover, the added value of a paid cadre is unknown in settings such as Tanzania where dispensaries are widespread and community access may not contribute to accessibility, quality, or acceptability of primary health care services. An extensive 2010 literature review of community health worker research revealed the diversity in the types of community health worker programs and substantial knowledge gaps [16]. There have been wide variations in training duration, content, and methods and inadequate attention to management, supervision, and career progression [17,22,27]. Additionally, availability of medicines, equipment, and transport are important determinants of effectiveness for service provision, which may also influence credibility among community members and motivation [17]. It is also hypothesized that community health worker programs could reduce system costs by shifting tasks to a cadre with shorter training and low remuneration as well as costs for the user by providing home-based care, but research on the cost-effectiveness is insufficient [18]. Motivation beyond recognition that financial remuneration is needed has also been under-researched [29].

A crucial component of any community health worker’s role is facilitating referrals [20,21] as a number of interventions to save the lives of mothers, newborns, and child require better access to facility-based care in emergencies and, hence, stronger referral mechanisms from the community [10-15] . For example, there is considerable evidence that emergency obstetric care (EmOC) reduces maternal and newborn mortality, however, inefficient functioning of referral systems often results in delayed treatment and poor outcomes [30]. A recent systematic review of evidence on referral interventions aimed at reducing maternal and newborn mortality found that many are ‘promising’ but conclusive evidence demonstrating their effect is scarce [31]. In addition, there are particular challenges in linking communities in moments of emergency (Figure 1).

The government of Tanzania has recently enacted policies aimed at continuing the long tradition of strengthening primary health care quality and access and the linkages between communities and health facilities. Tanzania’s Primary Health Services Development Programme (*Mpango wa Maendeleo wa Afya ya Msingi*, MMAM), calls for establishment of a dispensary in every village, a health center in every ward, and a cadre of community health workers and strengthening of referral systems [32]. The plan to introduce the new community health worker cadre is, however, still under development. Policy support and approval of the large-scale hiring of salaried community health workers awaits evidence of impact and sustainable costs. Efforts at improving referral have been piecemeal and face challenges in the essential linkage of community to facility.

To address this evidence gap, the Connect Project tests the hypothesis that introducing a formally trained and remunerated cadre of community health workers while improving district-level emergency referral systems will reduce child mortality. Additionally, it will improve access, utilization, and behaviors related to reproductive, maternal, newborn, and child health. Connect will also use quantitative and qualitative implementation data to provide operational guidance to the Ministry of Health and Social Welfare (MoHSW) in developing the community health worker cadre and strengthening emergency referral systems. Table 1 shows the core objectives of Connect.

**Table 1:** Connect Project intervention and research objectives

**Intervention** • To improve **equitable access** to routine and emergency MNCH services • To extend the **range** of MNCH services available in the community • To improve the **quality** of community-based and emergency MNCH services • To increase the **efficiency** of the health system to deliver community-based services and respond to health emergencies **Research** • Evaluate impact on infant and child health and nutrition and maternal, newborn and child mortality • Assess outcomes including service utilization and coverage, improved health behavior, increased equity and decreased social cost for health seeking behaviors • Assess implementation and operations in relation to service delivery and quality and health systems inputs/processes as well as individual, organizational, structural and contextual factors • Conduct cost-benefit analysis

A community-based cluster randomized trial design represents the most rigorous possible appraisal of the effectiveness of the community health agent (CHA) in the context of district health systems. The study is being implemented in three rural districts in Tanzania. The districts, and corresponding study villages, were selected to build upon previous and ongoing efforts to strengthen primary health care planning and facility services, as well as due to the presence of ongoing Health and Demographic Surveillance Systems (HDSS). The HDSS areas are comprised of 101 villages and 50 of those villages are randomly assigned to the CHA intervention. The village was chosen as the unit of randomization in order to align the design with the anticipated primary organizational unit of any national program that would recruit, train, and assign CHA to districts.

### Description of the intervention

**Figure 2:**
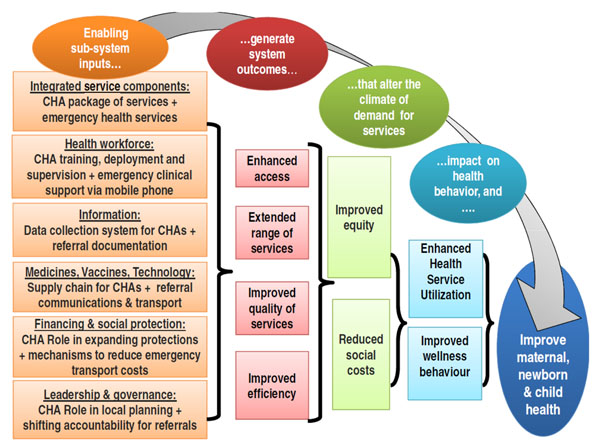
**Connect project theory of change.** The diagram illustrates the theory of change for the Connect project hypothesizing the effects that project health systems development inputs related to the CHA and Emergency Referral intervention components will have on systems' level outcomes and ultimately outcomes and impact at the population level.

The CHA introduction and activities to strengthen emergency referral constitute complex and interrelated interventions requiring multiple processes that correspond with the six building blocks of the health system proposed by the World Health Organization [33]. Figure 2 illustrates how the project hypothesizes that the inputs and activities across the six building blocks will ultimately leading to changes in population health. Although the diagram shows a linear progression from inputs to impact, Connect also aims to better understand the non-linear and unpredictable results as well as the multiple feedback loops that typically occur with the introduction of complex interventions into dynamic health systems [34].

#### Implementing partners

Connect is the result of a strategic partnership between Ifakara Health Institute (IHI) in Tanzania, Mailman School of Public Health at Columbia University (MSPH), the Tanzania Training Center for International Health (TTCIH), Council Health Management Teams (CHMT) in the three study districts, and the Connect Advisory Group (CAG), comprised of focal persons from the MoHSW, donor and multilateral partners, and civil society representatives. IHI is responsible for overall program design and implementation, including evaluation efforts, with technical assistance provided by MSPH. TTCIH led the design and implementation of the training program. Following an intensive initiation period, CHMTs from the three districts are incrementally undertaking more and more responsibility for implementation with support from IHI coordinators. The CAG convenes twice annually to review progress and results and provide advice on overcoming challenges.

#### Description of study sites

Across the three study districts, the census projections for 2010 estimate a total population of approximately 857,000 [35]. Just over half of the population is female (51.7%). In terms of age, 14.0% of the population are children under 5, while 28.5% are between 5-14 and 42.5% between 15-50. Of the total population, 362,307 reside within the Health and Demographic Surveillance Systems (HDSS) catchment area with HDSS populations for Kilombero, 197,393; Rufiji, 124,192; and Ulanga 40,722.

**Figure 3:**
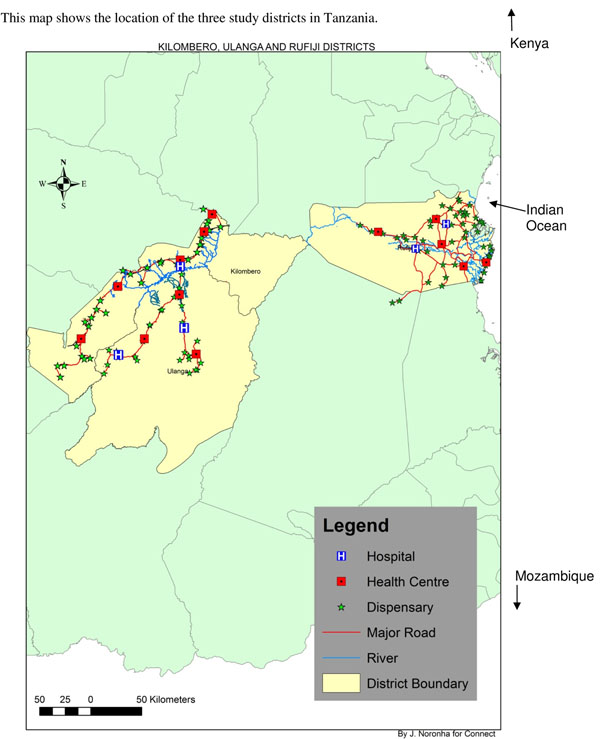
Connect project intervention areas.

Kilombero and Ulanga are isolated, impoverished districts that lie between 124-248 miles (200-400 km) from Dar es Salaam in the Morogoro Region of Tanzania (Figure 3). These areas are predominately rural and rely primarily upon subsistence agriculture. The Rufiji District is located in the Coastal Region and is also rural. Most inhabitants are subsistence farmers, though there is some trade in fishing and wood products. Transportation and infrastructure in all three districts are poor. The major causes of mortality in these districts are malaria, acute lower respiratory infections, tuberculosis, AIDS, and perinatal causes. Immunization coverage ranges from 85% for Bacillus Calmette-Guerin (BCG) vaccination to 66% for measles in children 12-23 months of age. Nearly 90% of the population in this study area lives within 5 km of a formal health facility [36-38].

Several district health systems strengthening programs have been implemented in these districts by the IHI. Most recently, these include the Tanzania Essential Health Interventions Project (TEHIP), which developed tools to facilitate evidence-based planning in districts; the Empower Project, which focuses on further strengthening district planning for maternal, newborn, and child health as well as facility-based MNCH services; and the ACCESS Project, which uses monitoring and rewards systems to motivate facilities to improve quality of care [36,39,40].

#### Community health worker intervention component – the CHA

The new cadre are called community health agents (CHA) to emphasize their role in facilitating linkages between the community and the health system. A CHA is defined as a health care worker who is formally trained and employed by the health system, provides a package of health services in the community, and connects people across the household to facility continuum. CHAs are members of, selected by, and accountable to, the communities where they work.

The CHA work package was inspired by the package of services provided through the community-based primary health care project in Navrongo, Ghana [41]. The Ghana PHIT partnership project [42] also informed development of the CHA job description, recruitment procedures, remuneration package, training curriculum, and supervision system. File 1 shows the range of services the CHAs are trained and authorized to provide. The main aspects of their services include 1) community-case management of under-5 childhood illness using IMCI principles; 2) ante- and post-natal pregnancy, delivery and essential newborn care counseling and danger signs identification; 3) family planning counseling and distribution; 4) and facilitation of emergency and routine referrals to facilities. CHAs are also engaged in encouraging enrollment in community-based insurance programs and identifying at-risk households. Additionally, they are linked to local governance structures, such as village health committees and facility governance committees, providing data for decision-making as well as advocating for community needs. (See Table 2 for a breakdown of the CHA work package.)

**Table 2:** CHA Work Package

Service	Description
***Health Promotion and Education***	

Behavior Change Communication	• Delivered through household visits and mobilization of men’s and women’s groups • Main topics are: family planning; antenatal care; delivery care; post-natal care; child health; HIV and STI prevention; water, sanitation and hygiene and environmental health.

***Focused Counseling and Service Delivery in Households***	

Family Planning	• Refer client to the nearest facility for family planning initiation • Make household visits to refill methods and provide counseling and support • Counsel and provide condoms and demonstrate their proper use

Antenatal Care	Three households visits during pregnancy: • Visit 1 at 12-16 weeks: counseling, promote early initiation of ANC within first 4 months, emergency referral in the case of complications; provision of condoms for HIV/STI prevention, monitor ITN use • Visits 2 and 3 at 20 and 28 weeks: promote 4+ ANC visits, provide emergency referral for complications, continue monitoring ITN use, IPTp and PMTCT, develop birth preparedness plans, and counsel on danger signs, essential newborn care, post-partum family planning and HIV prevention/PMTCT

Delivery Care	Emergency referral for obstetric and newborn complications: • Call for advice and transport • Mobilize community to create local solutions to the referral problem

Postnatal Care	Four visits in the postnatal period: • Visits 1 and 2 within 24 hours of delivery and 3 days after birth: ○ Intensified counseling on essential newborn care ○ Promotion of postpartum family planning ○ Refer for maternal and newborn complications ○ Measure the newborn’s foot size to detect low birth weight ○ Educate household on maternal and newborn postpartum danger signs • Visits 3 and 4 at 7 and 28 days to promote immunization and exclusive breastfeeding and refer for complications

Under-five Health	• Management of simple cases of diarrhea, pneumonia, malaria (pending RDT rollout), and helminthic infections; referral for severe cases of child illness • Mobilize villages and coordinate immunization and Vitamin A outreach events with health facility staff • Focused counseling on child nutrition, growth monitoring, and prevention and recognition of childhood illness • Depending on mothers’ voluntary disclosure, encourage HIV diagnosis of the newborn and infants at 18 months.

HIV/STIs and TB	• Provide HIV/STI prevention education and distribute condoms • Encourage VCT • Link PLWHA to support groups. • Identify potential cases of TB and refer

First Aid	• Perform first aid and refer

***Vulnerability and Risk Protection***	

Disability	• Identify individuals with disabilities • Refer to appropriate services

Social Protections	• Educate households on the Community Health Fund and encourage them to enroll • Identify poor households that qualify for exemptions

***Community Mobilization and Health Systems Strengthening***	

Mobilization and Outreach	• Convene village groups to identify and discuss priority community health issues • Mobilize community-based responses for emergency referrals and other priority issues

Health Information	• Collect service statistics (CHA) and assist with vital registration at village level • Manage and report community-based service information into broader HMIS

Governance and Leadership	• Attend Village Health Committee, Health Facility Management Team and Health Facility Governing Committee meetings to articulate community health needs and priorities • Support committees in coordinating events and activities as needed

The below table provides an overview of the main tasks CHA are trained and authorized to provide.

Utilizing all of the 101 villages under surveillance in the three districts, villages were randomly assigned to intervention (50) and comparison (51)*.* The number of CHA to serve in each of the intervention villages was determined as one for a population of <1,000; two for a population of 1,000 – 3,999; three for a population of 4,000 – 6,999; and four for a population of >7,000.

To recruit the CHA, local government solicited and screened applications and then convened a village meeting where qualified applicants were selected through voting. The minimum qualification was completion of a secondary education. Each CHA underwent a nine-month pre-service training in a residential competency-based program after which they were awarded a certificate that allows career progression. The training topics include human biology, community health, clinical skills, reproductive health and family planning, IMCI (including community case management), essential newborn care practices, disease prevention and control, and advocacy and communication. Due to training capacity, CHAs were trained in two cohorts with a total of 57 in the first round and 56 in the second round. All graduates were deployed to their home villages. A third cohort was trained in 2012-13 to increase the minimum number to two CHA per village based on early implementation feedback and to align work assignment according to official government decisions to create hamlets. Although the third round of training did not alter the experimental design, 32 CHA are being added to treatment villages to respond to decisions by the government of Tanzania to create administrative and political functions for hamlets within villages, which necessitates recalibration of CHA work areas according to this new political arrangement. After graduation, CHA are officially contracted and paid as employees of their local government with project funding.

System supports and linkages for the CHA are coordinated by a member of the CHMT. The supervision design is dual: nearby health facility staff provide supervision for technical issues and village governments provide supervision for administrative issues. Health facility supervisors hold a qualification equal or above that of an enrolled nurse. Village governments select a member of the community to serve as the village supervisor based on education level, age, residence in the village, and status in the community. Neither supervisor receives payment for their role as a CHA supervisor. The project provides them with mobile phones, job aids, and bicycles for village supervisors and motorbikes for supervising health facilities.

CHA-specific registers and reporting forms were developed for integration in district health information systems. The project also directly provides CHA with a specially designed kit of medicines, supplies and equipment, including a mobile phone and a bicycle. Efforts are under way to develop resupply mechanisms managed by the district governments utilizing project funding.

#### Emergency-referral intervention component

Despite ongoing programs, remaining weaknesses were identified in the district referral systems, thus, the project is supporting the development and implementation of locally designed strategies to strengthen district referral systems based on contextual evidence and the requisites for referral systems proposed by Murray and Pearson [29].

Because a whole systems approach was required, the referral intervention is being implemented at the district level. The design takes into account the required layers of coordination starting with prompt decision making at the household level; timely referrals from communities; appropriate triaging and pre-referral care; rapid transportation; and responsiveness at receiving facilities [43,44]. The project activities aim at improving the functioning of the system to respond to all emergency health conditions, with emphasis on obstetric, newborn, and child complications. These activities are linked to and draw upon the presence of the CHA to inform families about early warning signs of complications and facilitate early referrals, as well as to mobilize the development of community-based emergency mechanisms.

The intervention was informed by a mixed-methods formative research study conducted in the three districts. The research found that each district had planned and implemented some strategies and activities but none met the requisites of a strong referral system [29]. Furthermore, the responsibility for referral transport was typically borne by the woman and her family, resulting in delays and catastrophic expenditures. The formative research findings were used to inform the development of district-specific plans. These plans were developed through participatory action research and planning exercises that engaged stakeholders at district and community levels.

Strategies will be implemented by the districts with local resources as well as Connect financial and technical support. Funds mobilized by the districts will support advocacy with local politicians, upgrading health centers to provide emergency care, establishing hospital-based emergency teams, and including emergency care in supervision visits. Components supported by the project include improvements in ambulance transportation; establishment of an emergency telemedicine support, and advance communication system using mobile phones; clinical improvements in staff skills and patient flow for triage and pre-referral care; community mobilization mechanisms initiated and facilitated by CHA; and introduction of standardized emergency-referral forms.

### Evaluation design

The evaluation of the added value of the CHA is designed as a community-based cluster-randomized trial set in three HDSS villages to measure reductions in child mortality and outcomes related to reproductive, maternal, newborn, and child health. Table 3 presents examples of key outcomes of interest [45,46]. The district-wide emergency referral evaluation relies on facility and systems-based data. A plan of implementation research will complement this data, as well as an economic evaluation to assess the overall costs of the program.

**Table 3:** Examples of project maternal, newborn and child health outcomes of interest

Category	Indicator
**Mortality rates (by gender of child)**	• Neonatal mortality rate • Infant mortality rate • Under-age five cumulative mortality rate (q_5_ ) • Estimated under age 5 cause specific mortality rate for malaria, acute respiratory infections,

**Maternal Health Behavior (last live birth)**	• Percent of women who made their first ANC visit before the fourth month • Percent pregnant women who attended four or more antenatal care visits • Percent pregnant women who attended postnatal care visit • Percent women who deliver in a health facility • Proportion of c-sections among last born

**Newborn Health**	• Percent of newborns attending postnatal care visits • Percent of newborns breastfed within 1 hour of birth • Percent of women that report having practiced at least three ENC behaviors

**Under-5 child health**	• Percent episodes of diarrhea in children under 5 treated with ORS + zinc • Percent episodes of cough/pneumonia in children under age five treated with antibiotics • Percent of febrile malaria episodes among children under 5 treated within 24 hours of onset

**Family planning and fertility**	• Contraceptive prevalence rate • Unmet need for family planning • Age specific and total fertility rates

#### Ethical considerations

Ethical approval has been received from the institutional review boards of the IHI, the Tanzanian National Institute of Medical Research, the Tanzania Commission for Science and Technology, and Columbia University. Permission to conduct the intervention and corresponding research was obtained from relevant district and village government authorities. The trial has been registered with the International Standard Randomized Controlled Trial Register (#ISRCTN96819844). In addition, all reporting will follow CONSORT guidelines for cluster-randomized trials [47].

#### Evaluation of the CHA intervention

Villages were randomly assigned to either CHA intervention or comparison within four strata defined by village population size. This ensured that large and small villages would be equally represented in the intervention and control arms of the study. Village boundaries were identified from official government records. Intervention and comparison areas were tested for comparability in baseline mortality and key MNCH behavioral indicators. Analyses confirmed that the sample is balanced, with statistically insignificant differences between the two study arms for each metric tested.

A possible limitation of the design is the potential for clientele of comparison communities to seek care from CHA who are based in nearby treatment communities. To address this possible bias, research modules have been developed to assess comparison-area client exposure to CHA services. This information will permit statistical adjustment for cross-cell contamination. In addition, villages vary in distance to nearest health facility and health facility staffing. Some villages have a dispensary or health center and others are served by facilities outside their boundaries. Other biases could arise if project-provided supplies are differentially accessible to CHA relative to workers based in dispensaries. To address these biases, statistical adjustment will be made for distance to nearest health facility, health facility staffing, and disruption of key supplies at the dispensary level. Finally, an index of health systems strength developed will be included in multivariate models; CHA public health impact may vary according to the strength of the health systems in which CHA are placed.

Based on this information, Connect has developed a counterfactual basis for analyzing project impact on mortality, particularly measures of child mortality, and other measures of impact in comparison and intervention areas. Since Connect is a true experiment, and prior demographic trends are available, rigorous application of hazard regression models is possible. This permits the multilevel modeling of individual prospective longitudinal survival as a function of childhood, familial, community, and programmatic covariates; treatment indicators are adjusted for the population density of CHA exposure, distance-to-service points, and other confounders, such as maternal age and educational attainment, household distance from fixed service points, household relative economic status, and extended family size and composition [48]. The imposition of “time-conditional” models will permit analyses of the survival of all children who have ever been under 60 months of age during the Connect Project period.

Evaluating CHA impact on child mortality relies on the HDSS, a longitudinal data system which collects relational data from households three times annually. Mortality events are registered in conjunction with migration and births, providing a basis for monitoring the risk of childhood death. Conventional procedures for assessing cause of death will permit appraisal of maternal mortality and causes of child mortality [49]. In addition, HDSS modules in the study area have been added to assess proximate determinants of fertility, allowing for detailed analysis of the fertility impact of the CHA.

Hayes and Bennet’s formula published in 1999 for simple sample-size calculation for cluster-randomized trials was used to determine the level of power available to detect the effects of the intervention on health outcomes [50]. Connect embraced Van Breuklen et al’s approach to adjust estimates of statistical power for the loss of efficiency that arises from assigning CHA treatment in clusters, by village [51]. Based on these procedures, we have demonstrated that the study design has 80% power to detect 18% change in under-5 mortality, 20% change in infant mortality, and 25% change in newborn mortality.

Connect will use the detailed registration of demographic information at the individual level to closely monitor changes in intervention and comparison villages. Linked information on individuals, parents, households, or localities provides opportunities to model survival processes as a function of characteristics of their social or economic circumstances. Additionally, a household survey with a sample of approximately 3,300 women of reproductive age was conducted at baseline and will be conducted at endline in the study area to gather additional information on behavioral and care-seeking patterns. These data will permit individual-level analyses, providing a rich picture of relevant health seeking and service utilization trends and pathways at baseline and, by the end of the project, to explain the patterns that underlie survival impact, including patterns associated with care seeking and referral for maternal, newborn, and child emergencies.

#### Evaluation of emergency referral system strengthening

The emergency referral strengthening interventions cover the entire districts and address emergency health conditions among all members of the population, with a focus on pregnant women, newborns, and children under 5. Since only three districts are included in the design, randomization of the referral intervention was not possible and overall assessment of the referral component of Connect is constrained to a pre-post case study. To this end, data extraction from referral forms will be used to assess changes in volume and patterns of emergency referral systems [52,53]. Referral forms will also be used to establish whether changes occurred after introduction of the interventions [54].

Criterion-based audits on emergency-referral cases at baseline and endline will be used to assess changes in quality of management in these cases [55]. Health facility surveys, based on Service Provision Assessments and needs assessments for emergency obstetric care, will be conducted at baseline, midline, and endline for cross-sectional assessment of the relative strength of the health system, particularly as relates to maternal, newborn, and child health and ability to respond to health emergencies. The primary analysis of these data will develop a model that is used to generate measures of facility strength and preparedness for emergencies [56]. This method will provide data for assessing changes related to the provision and utilization of emergency services, particularly for acute maternal, newborn, and child health conditions. The facility survey will also provide data on the health system contextual factors that may affect CHA performance.

The referral system will be monitored in the 101 HDSS communities according to the pace and location of the introduction of CHA support for the referral system. Referral related demographic outcomes — such as maternal and early neonatal deaths in CHA exposed and unexposed communities — will be used to determine if the phasing in of referral has been helped with CHA support relative to having referral logistics without CHA implementation support. Connect will also conduct research using non-mortality referral system outcomes as endpoints. This will test whether the CHA role improves the emergency referral system since indicators will determine whether the referral system works better in villages with CHAs.

#### Implementation evaluation

To complement the impact and outcome evaluations, a parallel research agenda will examine the “black box” of mechanisms along the causal pathway that led to the observed changes [57]. This type of complementary research is increasingly recommended for trials of complex health systems interventions due to the challenges in assessing what contributed to the observed effects (or lack thereof) and the inherent dynamic nature of systems [58-60]. This complementary research will consist of documenting and analyzing the activities and processes (both planned and unplanned) that occurred to implement the intervention, including how, why, and by whom the processes occurred and how contextual factors both within and outside the formal health system influenced implementation. Quantitative and qualitative findings will be triangulated to provide a rich picture of the implementation processes and moderators and will permit a realistic evaluation of the Connect interventions [57,61,62]. Ongoing monitoring and feedback as part of this research will allow a process of refinement to improve implementation in collaboration with the CHMT, supervisors, and CHA.

To guide this research, a framework drawing from relevant literature was developed encompassing measures and concepts of the processes and potential moderators of implementation related to the intervention, the implementers, the system, and the context [63-66]. The framework includes measures of fidelity or adherence in implementing the intervention as per the design — an important potential mediator and moderator of the relationship between the designed intervention and the observed outcomes [67]. Measures are related to CHA service delivery but also to systems-level CHA supports and strengthening of referral. For the CHA services, for instance, adherence includes measures of whether services were delivered to the intended target populations (coverage), whether CHA provided the services intended (content), and how often they interacted with clients (frequency) [59,68]. These data will be captured through the HDSS, routine statistics from the CHA, the endline household survey, and assessments of CHA quality of care.

Potential implementation moderators will also be assessed through qualitative and quantitative methods. Qualitative methods will be used to explore concepts related to 1) the characteristics of the intervention, such as perceptions of its complexity and relative advantage over other interventions; 2) characteristics of individuals implementing the intervention, such as their perceived ability to perform the required tasks; 3) characteristics of the organizational setting, such as system priorities and leadership engagement; and 4) the responsiveness of the community in terms of acceptability of the intervention. Other contextual information will include village characteristics and relevant social, political, and economic events that occur during the course of implementation. “Qualitative systems appraisal,” a regimen comprised of focus group discussions and in-depth interviews, will be conducted at different levels of the health system every six months to capture these perceptions [69]. The performance, satisfaction, and motivation of the CHA and involved emergency referral health workers will also be analyzed utilizing quantitative methods. Periodic self-filled questionnaires and routine statistics will be used to capture these data.

## Discussion

To date, 113 CHA have been deployed to all 50 randomly selected intervention villages as planned. The emergency referral component, which is a district wide intervention, has completed the planning process and a number of initial activities, such as provision of transport and initiation of the emergency support telemedicine system. Based upon triangulation of monthly reports from field implementation coordinators, monthly statistics from CHA, examinations from training, and preliminary analysis of qualitative interviews and focus groups with stakeholders from multiple levels, we have drawn some initial implementation findings, which are described in Table 4. These areas are still under research and, as the study progresses, will be verified.

**Table 4:** Tanzania Connect Partnership project: Successes, Challenges, and Adaptations

***Successes***
**Development of a curriculum that creates the appropriate competencies** The results from the training showed that the recruits, with the specified qualifications, could be trained to properly apply the principles of community IMCI and manage uncomplicated cases of diarrhoea, pneumonia and malaria as well as provide a broader package of reproductive child health services. **Deployment and support of a new community-based cadre to provide RCH services** CHA have largely been able to provide the package of care in the real world context of their villages. Reports show that acceptability of the CHA and their services among a variety of stakeholders, including communities, health workers and district management, has been high. In addition, the supports designed for the CHA appear to have facilitated their work. **Collaboration and ownership by national, district and local government** At national level, processes are underway to define a new cadre of community health worker and Connect operational evidence has played an important role in these processes. In addition, the district and village authorities have been instrumental in facilitating the CHA introduction and mediating any problems or challenges encountered. The engagement for the referral planning has provided a solid foundation for ensuring the relevance of strategies to local conditions.

***Challenges***

**Chronic system shortages of medicines and supplies** There are nationwide supply chain challenges which include frequent stockouts of some essential medicines for RCH. As a result, the project has had to use vertical procurement mechanisms for the CHA. These stockouts also compromise the CHAs ability to provide added value to the system and to facilitate effective referrals and will affect the system’s ability to respond to emergency cases. **Effective supervision at the community level** The decision not to pay village supervisors to support the CHA, who are paid, has produced tensions that may have undermined this relationship. Village supervisors report both that they are doing more than requested and that they need more tools and motivation to do this work. **Balancing prevention, treatment and promotion services** Early findings reflect a widespread struggle among CHA to achieve a balance between the treatment and educational or promotional aspects of their role. This may reflect demand in addition to other factors as communities’ prioritized the provision of medicines more highly than other services.

***Adaptations***

**Coverage of CHA** Based on early implementation experience, areas served by one CHA were increased to two as many of these areas are large geographically even if populations are small. **Adaptations to the CHA kit** While the basic contents of the CHA kit were found to be largely appropriate, some medicines and supplies were later added based on feedback, for instance for first aid (e.g. iodine) and rapid diagnostic kits for malaria. **Extension of referral planning process** While it was expected that the planning process could be completed rapidly, while underway, additional stages were added to ensure that groups typically excluded from these processes, such as local transport drivers, were able to have a voice.

The Connect research design, in general, permits a unique opportunity to test the added value of a community health worker program on reducing child mortality, as well as improving behaviors and care seeking related to reproductive, maternal, newborn, and child health with a rigor that has been rare in similar studies in Africa [20]. The project uniquely tests the delivery of an integrated package of reproductive and child health services at the household level, rather than focusing on one particular disease or particular type of service. Connect is also designed to address key research priorities related to strengthening emergency referral systems for MNCH. By combining these two elements, Connect seeks to test operational interventions that close gaps in the “vertical” continuum of care from household to health facility [70,71]. Connect’s triangulation of demographic surveillance, cross-sectional panel surveys in households, and process evaluation will provide a rich picture of the programmatic and behavioral pathways that link program exposure, health systems strengthening, and changes in health outcomes and survival. These data are particularly important at a time when there are global calls to strengthen the continuum of care, including massive scale-up of community health workers in lower and middle income countries [68,72].

Connect represents a rigorously evaluated study that addresses the design limitations of other studies that have been fielded for assessing the added value of community-based primary health care workers in Africa [73]. In addition to Connect’s significance in advancing evidence for global and regional purposes, perhaps, most importantly, its links to national policy create an opportunity to provide evidence in support of efforts to reform how primary health care is delivered across Tanzania.

## List of abbreviations used

ANC: Antenatal care; BCG: Bacillus Calmette-Guerin; CAG: Connect Advisory Group; CHA: Community health agent; CHMT: Council Health Management Team; DHS: Demographic and health survey; EmOC: Emergency obstetric care; ENC: Essential newborn care; HDSS: Health and Demographic Surveillance System; IHI: Ifakara Health Institute; IMCI: Integrated management of childhood illness; MDG: Millennium Development Goal; MMAM: *Mpango wa Maendeleo wa Afya ya Msingi* (Swahili acronym for the Tanzanian government’s primary health services development program); MNCH: Maternal, newborn, and child health; MoHSW: Ministry of Health and Social Welfare; MSPH: Mailman School of Public Health; RCH: Reproductive child health; TEHIP: Tanzania Essential Health Interventions Project; TTCIH: Tanzanian Training Center for International Health; VHW: Village health worker; WHO: World Health Organization.

## Competing interests

The authors declare that they have no competing interests.

## Authors’ contributions

KR participated in the design and coordination of the study and drafted the manuscript. AH participated in the design and coordination of the study. MK led the baseline statistical analyses. EJ participated in the design of the study and led the randomization and sampling. AM participated in the randomization, sampling and baseline analyses. SP participated in the design of the CHA intervention and training program. FM designed the economic evaluation. CB contributed to the design of the study and drafting the manuscript. SH participated in the design of the study, sampling and baseline statistical analyses. JFP conceived of the study, and participated in its design and coordination and helped to draft the manuscript. All authors read and approved the final manuscript.

## Declarations

This article has been published as part of *BMC Health Services Research* Volume 13 Supplement 2, 2013: Improving primary health care to achieve population impact: the African Health Initiative. The full contents of the supplement are available online at http://www.biomedcentral.com/bmchealthservres/supplements/13/S2. Publication of this supplement was supported by the African Health Initiative of the Doris Duke Charitable Foundation.

## References

[B1] Jonsson U Ideological Framework and Health Development in Tanzania 1961-2000 Social Science in Medicine 22 7 745 753 3715514 10.1016/0277-9536(86)90226-1

[B2] Gilson L Alilio M Heggenhougen K Community satisfaction with primary health care services: An evaluation undertaken in the Morogoro region of Tanzania Social Science &Medicine 1994 39 6 767 780 10.1016/0277-9536(94)90038-8 7973873

[B3] Heggenhougen K Vaughan P Muhondwa EPY Rutabanzibwa Ngaiza J Community Health Workers: The Tanzanian Experience 1987 Oxford: Oxford University Press

[B4] Freeman P Perry HB Gupta SK Rassekh B Accelerating progress in achieving the millennium development goal for children through community-based approaches Global Public Health 2012 7 4 400 19 10.1080/17441690903330305 19890758

[B5] Perry H Freeman P Gupta S How effective is community-based primary health care in improving the health of children? 2009 Washington, DC: Health Care Working Group and American Public Health Association

[B6] Kruk ME Rockers PC Mbaruku G Paczkowski MM Galea S Community and health system factors associated with facility delivery in rural Tanzania: A multilevel analysis Health Policy 2010 97 2-3 209 16 10.1016/j.healthpol.2010.05.002 20537423

[B7] United Republic of Tanzania The National Road Map Strategic Plan to Accelerate Reduction of Maternal, Newborn and Child Deaths in Tanzania 2008 – 2015 2008 Dar es Salaam, Tanzania: Ministry of Health and Social Welfare

[B8] Masanja H deSavigny D Smithson P Schellenberg J Theopista J Mbuya C Upunda G Boerma T Victora C Smith T Mshinda H Child survival gains in Tanzania: analysis of data from demographic and health surveys The Lancet 2011 371 9620 1276 1283 18406862 10.1016/S0140-6736(08)60562-0

[B9] National Bureau of Statistics Tanzania ICF Macro Tanzania Demographic and Health Survey 2010 2011 Dar es Salaam, Tanzania

[B10] Shoo R Mzige A Review of community health worker cadres’ training programs in Tanzania Report commissioned by the United Republic of Tanzania Ministry of Health and Social Welfare and UNICEF 2011 Unpublished

[B11] Jones G Steketee RW Black RE Bhutta ZA Morris SS Bellagio Child Survival Study Group How many child deaths can we prevent this year? Lancet 2003 362 65 71 10.1016/S0140-6736(03)13811-1 12853204

[B12] Darmstadt GL Bhutta ZA Cousens S Adam T Walker N De Bernis L Lancet Neonatal Survival Steering Team Evidence-based, cost-effective interventions: how many newborn babies can we save? Lancet 2005 365 977 88 10.1016/S0140-6736(05)71088-6 15767001

[B13] Campbell OM Graham WJ Lancet Maternal Survival Series Steering Group Strategies for reducing maternal mortality: getting on with what works Lancet 2006 368 1284 99 10.1016/S0140-6736(06)69381-1 17027735

[B14] Bhutta ZA Ahmed T Black RE Cousens S Dewey K Giugliani E Haider BA Kirkwood B Morris SS Sachdev HP Shekar M Maternal and Child Undernutrition Study Group What works? Interventions for maternal and child undernutrition and survival Lancet 2008 371 417 40 10.1016/S0140-6736(07)61693-6 18206226

[B15] Glasier A Gülmezoglu AM Schmid GP Moreno CG Van Look PF Sexual and reproductive health: a matter of life and death Lancet 2006 368 1595 607 10.1016/S0140-6736(06)69478-6 17084760

[B16] PMNCH WHO Aga Khan University Essential interventions, commodities and guidelines for reproductive maternal, newborn, and child health 2011 Geneva: World Health Organization

[B17] WHO Global Health Workforce Alliance (GHWA) Global Experience of Community Health Workers for Delivery of Health Related Millennium Development Goals: A Systematic Review, Country Case Studies, and Recommendations for Integration into National Health Systems 2010 Geneva: World Health Organization

[B18] Lewin S Munabi-Babigumira S Glenton C Daniels K Bosch-Capblanch X Lay health workers in primary and community health care for maternal and child health and the management of infectious diseases Cochrane Database Syst Rev 2010 3 CD004015 20238326 10.1002/14651858.CD004015.pub3 PMC6485809

[B19] Phillips JF Bawah AA Binka FN Accelerating reproductive and child health programme impact: The Navrongo Experiment in Ghana Bulletin of the World Health Organization 2006 84 949 955 10.2471/BLT.06.030064 17242830 PMC2627578

[B20] Binka FN Bawah AA Phillips JF Hodgson A Adjuik M MacLeod B Rapid achievement of the child survival Millennium Development Goal: evidence from the Navrongo Experiment in northern Ghana Tropical Medicine and International Health 2007 12 5 578 593 10.1111/j.1365-3156.2007.01826.x 17445125

[B21] Gogia S Sachdev HS Home visits by community health workers to prevent neonatal deaths in developing countries: a systematic review Bulletin of the World Health Organization 2010 88 658 666B 10.2471/BLT.09.069369 20865070 PMC2930362

[B22] Haines A Sanders D Lehmann U Rowe A Lawn J Jan S Walker D Bhutta Z Achieving child survival goals: potential contribution of community health workers Lancet 2007 369 2121 31 10.1016/S0140-6736(07)60325-0 17586307

[B23] The CDI Study Group Community-directed interventions for priority health problems in Africa: results of a multi-country study Bulletin of the World Health Organization 2010 88 509 518 10.2471/BLT.09.069203 20616970 PMC2897985

[B24] Christopher JB Le May A Lewin S Ross DA Thirty years after Alma-Ata: a systematic review of the impact of community health workers delivering curative interventions against malaria, pneumonia and diarrhoea on child mortality and morbidity in sub-Saharan Africa Hum Resour Health 2011 9 27 10.1186/1478-4491-9-27 22024435 PMC3214180

[B25] Lee A Lawn JE Cousens S Kumar V Osrin D Linking families and facilities for care at birth: what works to avert intrapartum-related deaths? International Journal of Gynaecology and Obstetrics 2009 107 Suppl 1 S86 8 S65-S88 19815201 10.1016/j.ijgo.2009.07.012 PMC3428847

[B26] Habicht JP Victora CG Vaughan JP Evaluation designs for adequacy, plausibility and probability of public health programme performance and impact International Journal of Epidemiology 1999 28 1 10 18 10.1093/ije/28.1.10 10195658

[B27] Bhattacharya K Winch P LeBan K Tien M Community health workers incentives and disincentives: how they affect their motivation, retention and sustainability 2001 Arlington, Virginia: BASICS USAID

[B28] Walker DG Jan S The cost-effectiveness of community health workers: a review of the evidence and methodological critique J Community Health 2005 30 221 29 10.1007/s10900-004-1960-4 15847247

[B29] Strachan DL Kallander K Ten Asbroek AH Kirkwood B Meek SR Benton L Conteh L Tibenderana J Hill Z Interventions to improve motivation and retention of community health workers delivering Integrated Community Case Management (iCCM): stakeholder perceptions and priorities American Journal of Tropical Medicine and Hygiene 2012 87 Suppl 5 111 9 23136286 10.4269/ajtmh.2012.12-0030 PMC3748511

[B30] Murray S Pearson SC Maternity referral systems in developing countries: current knowledge and future research needs Soc Sci Med 2006 62 2205 2215 10.1016/j.socscimed.2005.10.025 16330139

[B31] Hussein J Kanguru L Astin M Munjanja S The effectiveness of emergency obstetric referral interventions in developing country settings: a systematic review PLoS Med 2012 9 7 e1001264 10.1371/journal.pmed.1001264 22807658 PMC3393680

[B32] United Republic of Tanzania Mpangowa Maendeleowa Afya ya Msingi (MMAM) 2007-2017 (Primary Health Services Development Programme) 2007 Dar es Salaam, Tanzania: Ministry of Health and Social Welfare

[B33] World Health Organization Everybody’s Business: Strengthening Health Systems to Improve Health Outcomes: WHO’s Framework for Action 2007 Geneva

[B34] deSavigny D, Adam T Systems Thinking for Health Systems Strengthening 2009 Geneva: Alliance for Health Policy and Systems Research, World Health Organization

[B35] National Bureau of Statistics Tanzania Population and Housing Census 2002 2006 Dar es Salaam, Tanzania: National Bureau of Statistics

[B36] Mwageni E Momburi D Juma Z Irema M Masanja H Rufiji DSS, Tanzania Population, Health, and Survival at INDEPTH Sites 2002 Ottawa: IDRC Press

[B37] Schellenberg JA Mukasa O Abdulla S Marchant T Lengeler C Kikumbih N Mshinda H Nathan R Ifakara DSS, Tanzania Population, Health, and Survival at INDEPTH Sites 2002 Ottawa: IDRC Press

[B38] Shabani J Lutambi AM Mwakalinga V Masanja H Clustering of under-five mortality in Rufiji Health and Demographic Surveillance System in rural Tanzania Global Health Action 2010 3 20838634 10.3402/gha.v3i0.5264 PMC2935925

[B39] Tanner M Strengthening district health systems Bulletin of the World Health Organization 2005 403 [serial online] PMC2626263 15976886

[B40] Hetzel MW Iteba N Makemba A Mshana C Lengeler C Obrist B Schulze A Nathan R Dillip A Alba S Mayumana I Khatib RA Njau JD Mshinda H Understanding and improving access to prompt and effective malaria treatment and care in rural Tanzania: the ACCESS Programme Malaria Journal 2007 6 83 doi:10.1186/1475-2875-6-83 10.1186/1475-2875-6-83 17603898 PMC1925101

[B41] Nyonator FN Awoonor-Williams JK Phillips JF Jones TC Miller RA The Ghana Community-based Health Planning and Services Initiative: fostering evidence-based organizational change and development in a resource-constrained setting Health Policy and Planning 2005 20 1 25 34 10.1093/heapol/czi003 15689427

[B42] Awoonor-Williams JK Bawah A Nyonator F Asuru R Oduro A Ofosu A Phillips J The Ghana Essential Health Interventions Program: a plausibility trial of the impact of health systems strengthening on maternal & child survival BioMed Central Health Services Research 2013 13 Suppl 1 S3 23819518 10.1186/1472-6963-13-S2-S3 PMC3668206

[B43] Kobusingye OC Hyder AA Bishai D Hicks ER Mock C Joshipura M Emergency medical systems in low- and middle-income countries: recommendations for action Bulletin of the World Health Organization 2005 83 626 631 16184282 PMC2626309

[B44] Razzak JA Kellermann AL Emergency medical care in developing countries: is it worthwhile? Bulletin of the World Health Organization 2002 80 11 900 5 12481213 PMC2567674

[B45] Gage AJ Disha A Suzuki C A Guide for Monitoring and Evaluating Child Health Programs 2005 Chapel Hill, North Carolina: MEASURE Evaluation

[B46] Bertrand J Escudero G Compendium of Indicators for Evaluating Reproductive Health Programs 2002 Chapel Hill, North Carolina: MEASURE Evaluation

[B47] Campbell MK Elbourne DR Altman DG CONSORT Group CONSORT statement: extension to cluster randomised trials BMJ 2004 328 7441 702 8 10.1136/bmj.328.7441.702 15031246 PMC381234

[B48] Bertrand M Duflo E Mullainthan S How much should we trust differences-in-differences estimates? Q J Econ 2004 119 249 275 10.1162/003355304772839588

[B49] Adjuik M Smith T Clark S Todd J Garrib A Kinfu Y Kahn K Mola M Ashraf A Masanja H Adazu K Sacarlal K Alam N Marra A Gbangou A Ewageni E Binka FN Cause-specific mortality rates in sub-Saharan Africa and Bangladesh Bulletin of the World Health Organization 2006 84 3 181 8 10.2471/BLT.05.026492 16583076 PMC2627285

[B50] Hayes RJ Bennett S Simple sample size calculation for cluster-randomized trials Int J Epidemiol 1999 28 319 326 10.1093/ije/28.2.319 10342698

[B51] van Breukelen GJ Candel MJ Berger MP Relative efficiency of unequal versus equal cluster sizes in cluster randomized and multicentre trials Stat Med 2007 26 2589 2603 10.1002/sim.2740 17094074

[B52] WHO UNFPA UNICEF AMDD Monitoring emergency obstetric care: A handbook 2009 Geneva: WHO

[B53] Murray S Davies S Phiri RK Ahmed Y Tools for monitoring the effectiveness of district maternity referral systems Health Policy Plan 2001 16 4 353 361 10.1093/heapol/16.4.353 11739360

[B54] Kumar V Kumar A Das V Srivastava NM Baqui AH Santosham M Darmstadt GL Community-driven impact of a newborn-focused behavioral intervention on maternal health in Shivgarh, India IntJ Gynaecol Obstet 2012 117 1 48 55 10.1016/j.ijgo.2011.10.031 22281244

[B55] Strand RT de Campos PA Paulsson G de Oliveira J Bergström S Audit of referral of obstetric emergencies in Angola: a tool for assessing quality of care Afr J Reprod Health 2009 13 2 75 85 20690251

[B56] National Bureau of Statistics [Tanzania] Macro International, Inc Tanzania Service Provision Assessment Survey 2006 2007 Dar es Salaam, Tanzania: National Bureau of Statistics and Macro International, Inc

[B57] Karachi TW Abbott RD Catalano RF Haggerty KP Fleming CB Opening the black box: using process evaluation measures to assess implementation and theory building American Journal of Community Psychology 1999 27 5 711 31 10.1023/A:1022194005511 10676545

[B58] Taghreed A Hsu J de Savigny D Lavis J Røttingen JA Bennett S Evaluating health systems strengthening interventions in low-income and middle-income countries: are we asking the right questions? Health Policy and Planning 2012 27 iv9 iv19 10.1093/heapol/czs086 23014156

[B59] Hawe P Shiell A Riley T Gold L Methods for exploring implementation variation and local context within a cluster randomised community intervention trial J Epidemiol Community Health 2004 58 788 793 10.1136/jech.2003.014415 15310806 PMC1732876

[B60] Oakley A Strange V Bonell C Allen E Stephenson J RIPPLE Study Team Process evaluation in randomised controlled trials of complex interventions BMJ 2006 332 7538 413 416 10.1136/bmj.332.7538.413 16484270 PMC1370978

[B61] Pawson R Tilley N Realistic Evaluation 1997 London: Sage Publications Limited

[B62] Lewin S Glenton C Oxman AD Use of qualitative methods alongside randomised controlled trials of complex healthcare interventions: methodological study BMJ 2009 339 b3496 10.1136/bmj.b3496 19744976 PMC2741564

[B63] Carroll C Patterson M Wood S Booth A Rick J Balain S A conceptual framework for implementation fidelity Implementation Science 2007 2 40 10.1186/1748-5908-2-40 18053122 PMC2213686

[B64] Damschroder LJ Aron DC Keith RE Kirsh SR Alexander JA Lowery JC Fostering implementation of health services research findings into practice: a consolidated framework for advancing implementation science Implement Sci 2009 4 50 10.1186/1748-5908-4-50 19664226 PMC2736161

[B65] Fajans P Simmons R Ghiron L Helping public sector health systems innovate: the strategic approach to strengthening reproductive health policies and programs American Journal of Public Health 2006 96 3 435 40 10.2105/AJPH.2004.059907 16449594 PMC1470499

[B66] Greenhalgh T Robert G MacFarlane F Bate P Kyriakidou O Diffusion of innovations in service organizations: systematic review and recommendations Milbank Q 2004 82 4 581 629 10.1111/j.0887-378X.2004.00325.x 15595944 PMC2690184

[B67] Fixsen D Naoom SF Blase DA Friedman RM Wallace F Implementation Research: A Synthesis of the Literature 2005 Tampa, FL: University of South Florida, Louis de la Parte Florida Mental Health Institute, The National Implementation Research Network FMHI Publication #231

[B68] Steckler A Linnan L Process Evaluation for Public Health Interventions and Research 2002 San Francisco: Jossey-Bass

[B69] Nyonator F Jones TC Miller RA Phillips JF Awoonor-Williams J Guiding the Ghana community-based health planning and services approach to scaling up with qualitative systems appraisal International Quarterly of Community Health Education 2004 23 3 189 213 (Retrieved from http://ezproxy.cul.columbia.edu/login?url=http://search.proquest.com/docview/61366923?accountid=10226.)

[B70] Kerber KJ de Graft-Johnson JE Bhutta ZA Okong P Starrs A Lawn JE Continuum of care for maternal, newborn, and child health: from slogan to service delivery Lancet 2007 370 1358 69 10.1016/S0140-6736(07)61578-5 17933651

[B71] Bahl R Qazi S Darmstadt GL Martines J Why is continuum of care from home to health facilities essential to improve perinatal survival? Semin Perinatol 2010 34 477 485 10.1053/j.semperi.2010.09.001 21094421

[B72] Ki-Moon B United Nations Secretary-General’s Global Strategy for Women’s and Children’s Health 2010 New York: United Nations

[B73] Handlos LN Chakraborty H Sen PK Evaluation of cluster-randomized trials on maternal and child health research in developing countries Tropical Medicine and International Health 2009 14 8 947 956 10.1111/j.1365-3156.2009.02313.x 19563429

